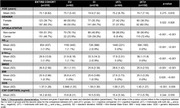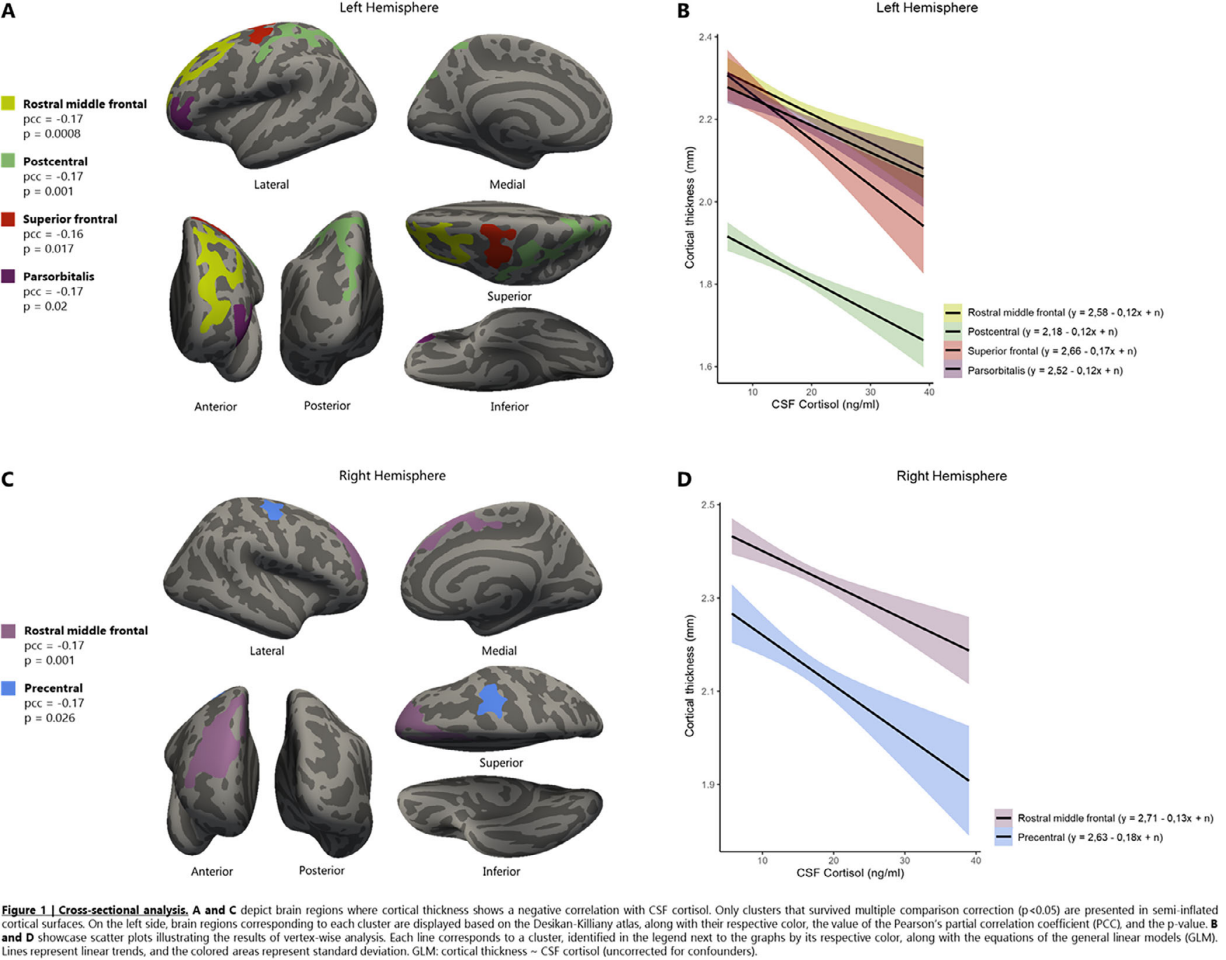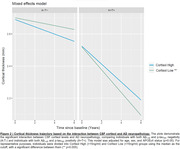# Association of CSF cortisol with cortical thickness in Alzheimer's disease continuum

**DOI:** 10.1002/alz.093312

**Published:** 2025-01-09

**Authors:** Laura Willers Souza, Lucas Uglione Da Ros, Andrei Bieger, João Pedro Ferrari‐Souza, Wyllians Vendramini Borelli, Marco Antônio de Bastiani, Guilherme Bauer‐Negrini, Jonathan M. DuBois, Eduardo R. Zimmer

**Affiliations:** ^1^ Universidade Federal do Rio Grande do Sul, Porto Alegre, Rio Grande do Sul Brazil; ^2^ Universidade Federal Do Rio Grade Do Sul, Porto Alegre, Rio Grande do Sul Brazil; ^3^ University of Pittsburgh, Pittsburgh, PA USA; ^4^ Departments of Psychiatry and Neurology, University of Pittsburgh School of Medicine, Pittsburgh, PA USA; ^5^ Memory Center, Hospital Moinhos de Vento, Porto Alegre, RS Brazil; ^6^ Clinical Hospital of Porto Alegre, Porto Alegre, Rio Grande do Sul Brazil; ^7^ Brain Institute of Rio Grande do Sul (InsCer), PUCRS, Porto Alegre, Rio Grande do Sul Brazil; ^8^ Biogen, Cambridge, MA USA; ^9^ Universidade Federal do Rio Grande do Sul, Porto Alegre Brazil; ^10^ Brain Institute of Rio Grande Do Sul, PUCRS, Porto Alegre, RS Brazil; ^11^ McGill Centre for Studies in Aging, Montreal, QC Canada

## Abstract

**Background:**

Multiple studies have linked high cortisol levels, a frequently used biomarker of stress, with the Alzheimer's disease (AD) pathophysiology. However, the relationship between cerebrospinal fluid (CSF) cortisol levels and AD‐related brain atrophy is not fully understood. This study sought to investigate the cross‐sectional and longitudinal association between CSF cortisol levels and brain cortical thickness in patients across the biological and clinical continuum of AD.

**Methods:**

We evaluated 310 individuals from the ADNI cohort with available baseline CSF cortisol concentrations, structural MRI and CSF Elecsys biomarkers (Aβ1‐42 and p‐tau181). Cross‐sectional analysis was conducted in Freesurfer (v7.1.1) through vertex‐wise analysis using general linear models (GLMs) corrected for multiple comparisons through cluster formation (p<0.01) and permutation (Monte Carlo simulation of 10,000 iterations). Longitudinal measures of an AD signature meta‐ROI were estimated from the surface area‐weighted average of the mean cortical thickness of the following ROIs: entorhinal, inferior temporal, middle temporal, and fusiform. Linear mixed‐effects (LME) models corrected for confounders were performed to evaluate cortical thickness longitudinal trajectories. The following models were used to assess the association between cortical thickness and cortisol levels: (1) cortisol as an independent variable; (2) cortisol, clinical diagnoses, and interaction; (3) cortisol, AD biomarkers positivity, and interaction.

**Results:**

Demographics are depicted in Table 1. Cross‐sectional analysis revealed that higher cortisol levels were associated with higher brain atrophy in several brain regions, as depicted by cortical brain thickness measures (Figure 1). These analyses also showed that cortisol does not interact with clinical diagnoses or AD biomarkers to decrease cortical thickness. In the longitudinal analyses, the association between cortisol and cortical thickness and the interaction with clinical diagnoses were not significant. However, we found that cortisol significantly interacts with AD biomarkers positivity to reduce cortical thickness values over time (β=0.002, p=0.03; Figure 2).

**Conclusions:**

Altogether, our results support that cortisol levels potentiate AD pathophysiology effects on AD‐related brain atrophy. These findings suggest that CSF cortisol levels may affect brain vulnerability to AD pathophysiology in the long term through neurodegeneration. Thus, our results have potential implications for understating AD‐related brain atrophy and developing innovative therapeutic strategies.